# Aggressive Natural Killer Cell Leukemia in an Adolescent Patient: A Case Report and Literature Review

**DOI:** 10.3389/fped.2022.829927

**Published:** 2022-05-23

**Authors:** Rong Yang, Yuan Ai, Chuan Liu, Xiaoxi Lu

**Affiliations:** ^1^Key Laboratory of Birth Defects and Related Diseases of Women and Children, Ministry of Education, West China Second University Hospital, Sichuan University, Chengdu, China; ^2^Department of Pediatrics, West China Second University Hospital, Sichuan University, Chengdu, China; ^3^Department of Radiology, West China Second University Hospital, Sichuan University, Chengdu, China

**Keywords:** aggressive natural killer cell leukemia, children, Epstein-Barr virus, hemophagocytic lymphohistiocytosis, case report

## Abstract

Aggressive natural killer cell leukemia (ANKL) is a rare malignant tumor, especially uncommon in children. ANKL has very aggressive clinical course and bad prognosis and is usually caused by Epstein-Barr virus infection. ANKL often has clinical manifestations of hemophagocytic lymphohistiocytosis (HLH) and can be easily treated as HLH, which might complicate this aggressive disease. Here we report an ANKL in adolescent whose clinical presentation was highly aggressive and response to L-asparaginase containing chemotherapy was very bad. Early-onset Flow cytometry of peripheral blood and bone marrow help make the diagnosis.

## Introduction

Aggressive natural killer cell leukemia (ANKL) is classified as a mature NK cell malignant tumor in the 2017 WHO classification of hematopoietic and lymphoid tumor (1). The most prominent feature of ANKL is its high aggressive clinical course and bad prognosis, with the median survival of less than 2 or 3 months (2, 3). Here we report a case of ANKL in adolescent with aggressive clinical course and central nervous system involvement. We gave the patient L-asparaginase containing chemotherapy but the patient showed poor response to it and experienced recurrent fever, deteriorated bleeding tendency, persistent disseminated intravascular coagulation (DIC), pancytopenia and liver dysfunction. Finally, the patient deceased due to lethal hemorrhagic complications. In this study, we draw the whole clinical course of the patient and review the literature of ANKL.

## Case Report

**FIGURE 1:**
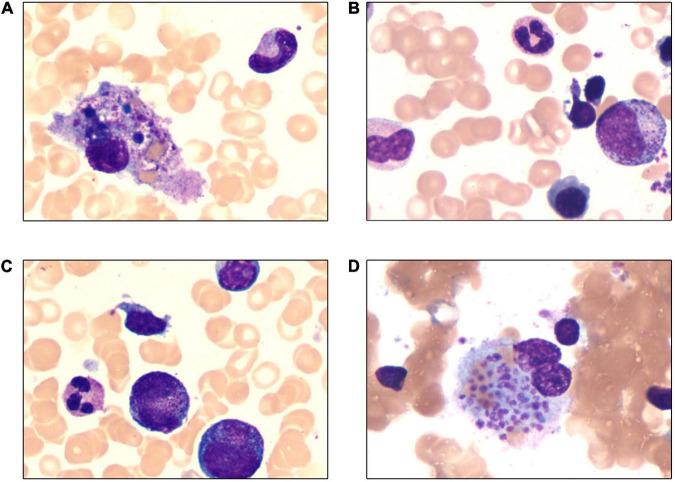
Wright-Giemsa stain of bone marrow (×1,000). **(A)** Hemophagocytosis in bone marrow smear. **(B–D)** Large granular variant lymphocytes in bone marrow smear and the proportion was 6%.

**FIGURE 2:**
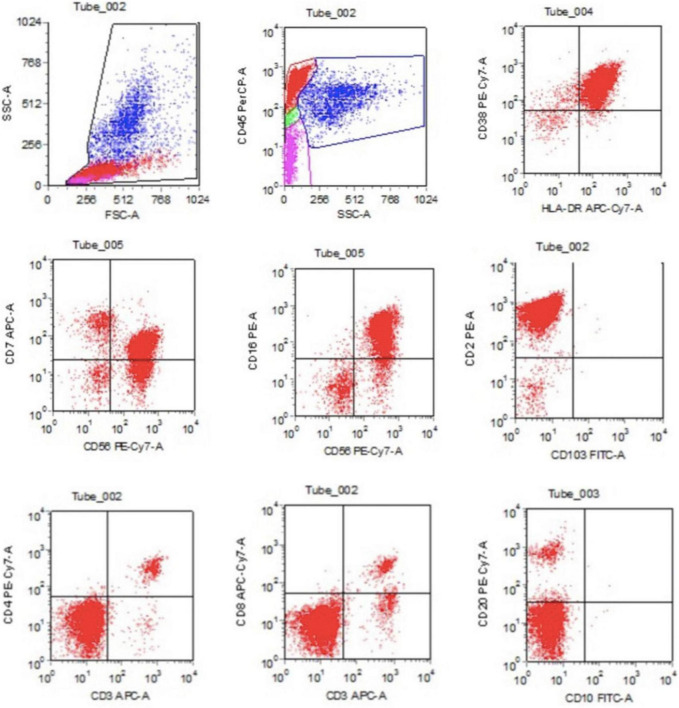
The flow cytometry detection of bone marrow revealed CD3–CD56+ cells counted for 55.67% of the lymphocytes, with HLA-DR+, CD2+, CD7 (dim)+, CD16+, CD38+, CD56+, and CD3–.

**FIGURE 3:**
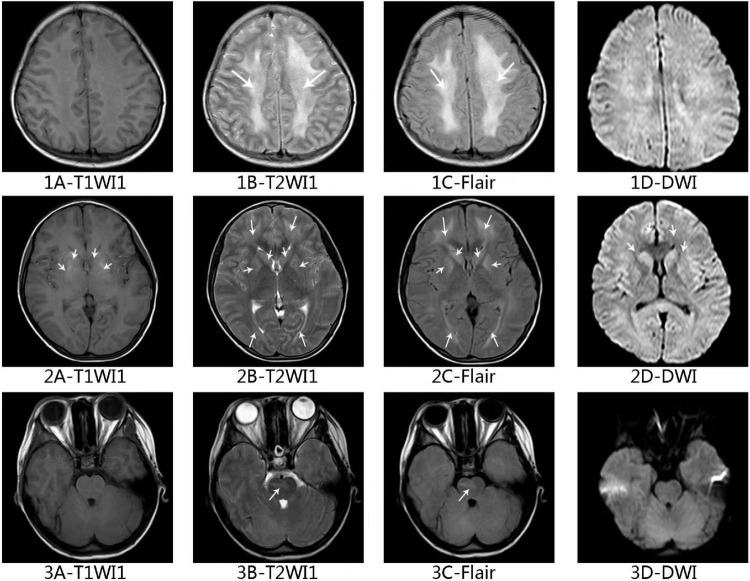
Axial magnetic resonance imaging of the brain in this ANKL patient. Axial T2-weighted (1B–3B) and fluid-attenuated inversion recovery (1C–3C) of brain showing white matter high signal at bilateral centrum ovale, periventricular areas, basal ganglia areas and pontine. Axial T1-weighted (2A) imaging and diffusion-weighted imaging (2D) shows high signal at areas of putamen and caudate nucleus head.

A 12-year-old female with no prior medical history was admitted to PICU because of fever for 4 days and disturbance of consciousness. The admitting physical examination revealed her in a coma with positive meningeal irritation sign and Babinski sign. Hepatomegaly, splenomegaly, hemorrhagic spots and ecchymosis were detected when she was admitted. The complete blood count revealed pancytopenia (WBC 4.0 × 10^9^/L, Lymphocyte 64%, Neutrophil 30%, atypical lymphocyte 4%, Hb 70 g/L, and PLT 54 × 10^9^/L). Large granular variant lymphocytes were identified in the peripheral blood smear. Blood biochemistry found increased ALT, AST, and LDH (ALT 417 U/L, AST 478 U/L,

and LDH 2,150 U/L). Blood coagulation function test revealed extension of APTT (41.1 S) and decrease of fibrinogen (Fg) (88 mg/dL). Ferritin was found significantly increased in the peripheral blood (1357.4 ng/ml) and Epstein-Barr virus (EBV) DNA was 2.5 × 10^5^ copies/ml in plasma. Hemophagocytosis and large granular variant lymphocytes were scattered seen in bone marrow smear, and the proportion of large granular variant lymphocytes was 6% (Figure 1). Flow cytometry testing (FCM) of the peripheral blood revealed significant increase of CD3–CD56+ lymphocytes, counted for 75.57% of the white blood cells. This group of cells also expressed CD2, CD7, CD16, and HLA-DR. FCM of bone marrow revealed CD3–CD56+ cells counted for 55.67% of the lymphocytes, with HLA-DR+, CD2+, CD7 (dim)+, CD16+, CD38+, CD56+, and CD3- (Figure 2). Chromosome karyotype analysis found 46, XX, t (6;9) (q23; p24). Next generation sequencing (NGS) of bone marrow showed splice site heterozygous mutations in TET2 gene (c.4045-1G>A), which was confirmed as somatic acquired mutation. Whole transcriptome sequencing (WTS) revealed two fusion genes positive: TPM4–KLF2 and EIF4A1–EIF5A. Protein level in cerebrospinal fluid was significantly increased (2,441 mg/L) and cerebrospinal fluid cytology found nucleated cells count was 20 × 10^6^/L, with 99% lymphocytes. Head magnetic resonance imaging (MRI) showed white matter high signal at bilateral centrum ovale, periventricular areas, basal ganglia areas and pontine. Axial T1-weighted imaging and diffusion-weighted imaging showed high signal at areas of putamen and caudate nucleus head (Figure 3).

The patient had no prior infectious medical history and we did not consider immunodeficiency of her. She was diagnosed as EBV associated hemophagocytic lymphohistiocytosis (EBV-HLH) initially and was treated with HLH-94 regimen for 3 weeks, including one-time intrathecal chemotherapy (4). The patient’s state of consciousness was improved but she showed recurrent fever, hematochezia and ecchymosis appeared all over the body. About 2 weeks after treatment, she was complicated with liver dysfunction and disseminated intravascular coagulation (DIC) (ALT 3,023 U/L, AST 2,146 U/L, conjugated bilirubin 3 mg/dl, PT 15.1 s, APTT 56.9 s). We gave her two times plasma exchange therapy, with no improvement of conjugated bilirubin, PT and APTT.

**FIGURE 4:**
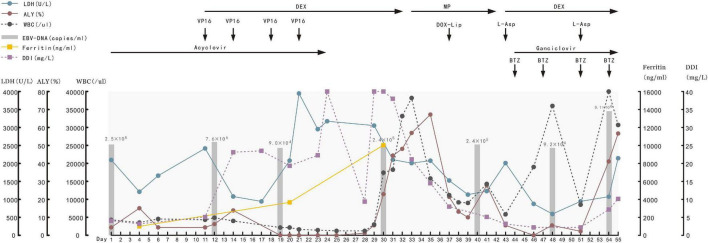
Typical laboratory indexes of blood and the drugs used for the patient from day of admitting, she died 55 days later. LDH, lactic dehydrogenase; ALY, atypical lymphocyte; WBC, white blood cell; DDI, D-Dimer; DEX, dexamethasone; MP, methylprednisolone; VP16, etoposide; DOX-lip, doxorubicin liposomes; L-Asp, L-asparaginase; BTZ, bortezomib.

Based on the presentation of recurrent fever, DIC, hepatic dysfunction, and HLH, EBV-DNA positive, large granular variant lymphocytes in bone marrow, significant increase of CD3–CD56+ lymphocytes in peripheral blood and bone marrow, diagnosis of ANKL was rendered (2, 5). We gave the patient L-DEP (L-asparaginase, doxorubicin liposomes, etoposide and methylprednisolone/dexamethasone) as the salvage regimen (6, 7). Considering persistent positive EBV-DNA, we gave the patient bortezomib plus ganciclovir for antivirus treatment (8), which was administered after the acquisition of approval from the Committee on Pharmaceutical Administration and Therapeutics and the consent of the patient’s parents. Despite high-intensive chemotherapy and strong supportive treatment, the patient still experienced recurrent fever, deteriorated bleeding tendency, persistent DIC and pancytopenia, EBV-DNA copies and plasma ferritin level even increased. After 4 weeks of chemotherapy, FCM of bone marrow revealed CD3–CD56+ cells increased to 84%. Ultimately this patient deceased due to lethal hemorrhagic complications: pneumorrhagia and gastrointestinal bleeding (Figure 4).

## Discussion

Aggressive natural killer cell leukemia is a rare malignant tumor and about 400 cases reported (5) (Supplementary Table 1). ANKL is much more common in East Asia and prone to occur in adolescents and young adults (AYA) (9). The patient’s condition is often critical, with fever, liver dysfunction, DIC and HLH (9–16). ANKL can be easily treated as HLH initially because it usually has very typical clinical manifestations of HLH but no unique clinical symptoms itself. Actually, diagnosis criteria of HLH cannot tell primary or secondary HLH and HLH-directed therapy may delay critical opportunities for diagnosis and curative treatment of the underlying pathology (17). ANKL is just one of the malignant triggers of HLH and we emphasize the necessity of identifying pathogenic lesions of HLH. Early recognition and prompt management are important to improve outcomes.

There are no unified diagnostic criteria for ANKL. Morphology and immunology features of peripheral blood and bone marrow are essential to make diagnosis of ANKL (18). Typical large granular lymphocytes could be found in bone marrow and peripheral blood, and hemophagocytosis by monocyte-macrophages could be observed in bone marrow smear (9, 13). ANKL should be differentiated to other large granular lymphocytic leukemia (LGL), especially T-cell granular lymphocytic leukemia (T-LGL). T-LGL shows a mature post-thymic phenotype with CD3+, TCRαβ+, CD8+, CD16+, CD45RA+, CD57+, CD4−, CD27−, CD28−, and CD45RO− (19, 20). Immunophenotype of ANKL suggests the proliferative lymphocytes originate from NK cells, that is CD56+, CD16+, CD2+, CD7+, sCD3−, CD4−, and no TCR arrangement (9, 10, 13, 14, 21). Frequent chromosome abnormalities reported in ANKL include 7p^–^, 17p^–^, and 1q^+^ (22). Cytogenetic analysis reveals complex abnormalities were common in ANKL patients, which involves three or more abnormalities of chromosome (10, 23). Next generation sequencing (NGS) found frequent genetic mutations in JAK/STAT signaling pathway, cell cycle regulation and DNA damage repair, epigenetic modifiers, RAS-MAPK signaling pathway, RNA helicase and mRNA splicing (23–25). JAK/STAT signaling pathway might be potential therapeutic target but further research needed (25).

Epstein-Barr virus (EBV) is considered contribute to the pathogenesis of ANKL (26–28) and EBV positive ANKL patients are more likely to have multi-organ involvement and are more likely to have HLH (9). Initial study considers EBV-negative ANKL patients have longer survival and are more likely to achieve complete remission compared with EBV-positive ANKL (29), while recent reports suggest EBV-negative patients have similar fulminant and aggressive clinical course (11, 30, 31).

The NK tumor cells can produce P-glycoproteins and it cause poor treatment response to cyclophosphamide, doxorubicin, vincristine and prednisone (32, 33). Study shows L-asparaginase can induce apoptosis of natural killer-cell tumors (34) and L-asparaginase-containing regimen is recommended for ANKL, such as SMILE (dexamethasone, methotrexate, ifosfamide, L-asparaginase, and etoposide), AspaMetDex (L-asparaginase, methotrexate, and dexamethasone) and VIDL (etoposide, ifosfamide, dexamethasone, and L-asparaginase) (11, 35). However, after L-asparaginase-containing chemotherapy less than 20% could achieve complete remission and they need hematopoietic stem cell transplantation (HSCT) to improve outcome (11, 35–39). According to the International Blood and Marrow Transplant Research database, achieving CR before HSCT appeared to be a key determination of successful outcome (38). The 2-year estimates of non-relapse mortality, relapse/progression, progression free survival (PFS), and overall survival (OS) were 21, 59, 20, and 24% (38). Studies have reported potential novel therapeutic applications, such as BCL2 inhibitors (25) and Heat Shock Protein 90 inhibitors, however, additional investigations are needed to determine whether they are effective.

The patient we report was admitted in PICU and the initial diagnosis was EBV-HLH. The diagnosis of ANKL was not established until 2 weeks later. The very first important clue was the increased NK cell proportion in lymphocyte subsets classification test, which was value by hematologist consultation. Than the FCM showed significant increase of CD3–CD56+ lymphocytes in peripheral blood and bone marrow, which helped making the ANKL diagnosis. The lesson is EBV-HLH might not be the final diagnosis and the possible underlying disease still needs to be tracking. Clinicians of ICU and hematology should be aware of it. The patient was in very bad general condition and poor response to the L-asparaginase containing chemotherapy, with recurrent fever, deteriorated bleeding tendency, persistent DIC and pancytopenia. The patient had disturbance of consciousness when admitted and head MRI showed white matter high signal. There are less than five cases of CNS involvement in ANKL reported (36, 40). Current evidence shows poor prognosis of CNS involved NK/T cell neoplasm (41). The head MRI of this patient was familiar with reported neuroimaging features in HLH patients (42, 43). TET2 gene mutation in this patient was confirmed somatic acquired and TET2 mutation has been reported in ANKL (23–25), AML, MPN, MDS, and CMML (44–46), associating with bad prognosis. Fusion gene TPM4–KLF2 was reported in ALL but the clinic significance was unclear (47–49). Fusion gene EIF4A1–EIF5A has not been reported but there are reports about the tumorigenic function of EIF4A1 and EIF5A (50–52). More study of these two fusion genes needed on their function of blood malignancy. Therefore, the ANKL we report had several unfavorable prognostic factors: bad general condition, CNS involvement, TET2 mutation, and poor response to chemotherapy and she finally died of lethal hemorrhagic complications.

In conclusion, it is very important to find the possible underlying lesions of HLH. For the HLH patients who has highly aggressive clinical manifestations, we recommends early-onset bone marrow smear, FCM of peripheral blood and bone marrow to figure out if there are increased NK cells. Doctors should control the lethal bleeding complication of ANKL. L-Asparaginase-containing chemotherapy might help achieve complete remission and the patient should prepare allo-HSCT earlier. Further researches of ANKL are needed to find novel cure for this deadly disease.

## Data Availability Statement

The original contributions presented in the study are included in the article/Supplementary Material, further inquiries can be directed to the corresponding author.

## Ethics Statement

Written informed consent was obtained from the minor(s)’ legal guardian/next of kin for the publication of any potentially identifiable images or data included in this article.

## Author Contributions

RY, YA, CL, and XL conceptualized and designed the study, drafted the initial manuscript, reviewed, revised, approved the final manuscript as submitted, and agreed to be accountable for all aspects of the work.

## Conflict of Interest

The authors declare that the research was conducted in the absence of any commercial or financial relationships that could be construed as a potential conflict of interest.

## Publisher’s Note

All claims expressed in this article are solely those of the authors and do not necessarily represent those of their affiliated organizations, or those of the publisher, the editors and the reviewers. Any product that may be evaluated in this article, or claim that may be made by its manufacturer, is not guaranteed or endorsed by the publisher.
