# Supplementary material for: Aggressive Natural Killer Cell Leukemia in an Adolescent Patient: A Case Report and Literature Review
Source: Front Pediatr. 2022 May 23;10:829927. doi: 10.3389/fped.2022.829927 (PMC9168658; doi:10.3389/fped.2022.829927)
Supplement: Supplementary file 1 [file Data_Sheet_1.pdf]

Supplementary Material

Supplementary Figures and Tables

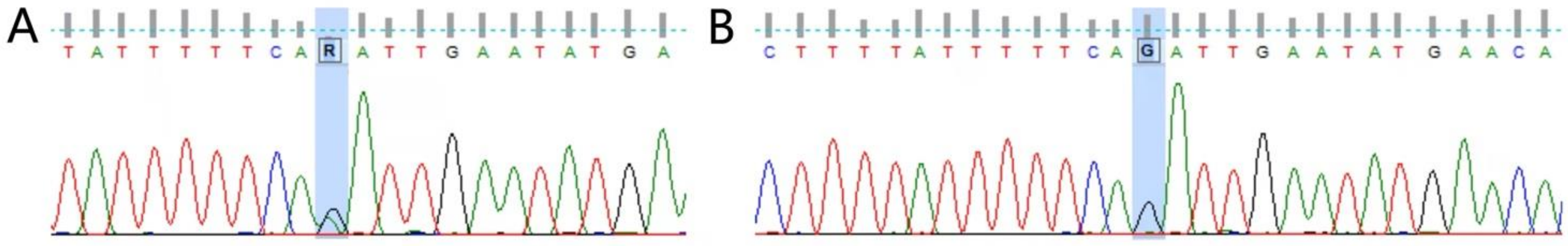

Supplementary Figure 1 Next generation sequencing of bone marrow showed splice site heterozygous mutations in TET2 gene, which was confirmed as somatic acquired mutation. (A)Splice site heterozygous mutation in TET2 gene of BM (c.4045-1G＞). (B) No mutation found in TET2 gene of oral mucosa.

Supplementary Table 1 Summary of ANKL reported in literature

Country	Year	Auther	PatientsNumber	Age median (range)	Mortality	mOS (days)
Japan	1990	Imamura et al(1)	4	20(13-30)	100%	450
Korea	2002	Song et al(2)	13	42(19-64)	100%	47
New Zealand	2004	Ruskova et al(3)	5	53(32-62)	100%	78
Japan	2004	Suzuki et al(4)	22	42(12-80)	95.4%	58
China	2006	Ryder et al(5)	9	45(22-70)	100%	50
Japan,Korea	2012	Ishida et al(6)	34	40(16-76)	94.1%	51
China	2014	Zhang et al(7)	20	40(17-67)	100%	56
China	2016	Li et al(8)	47	30(14-65)	95.7%	27
Korea	2016	Jung et al(9)	21	50(16-75)	71.4%	210
America	2017	Nicolae et al(10)	7	63(22-83)	85.7%	133
China	2017	Tang et al(11)	113	37(10-78)	95.6%	55

Supplementary references:

1. Imamura N, Kusunoki Y, Kawa-Ha K, Yumura K, Hara J, Oda K, et al. Aggressive natural killer cell leukaemia/lymphoma: report of four cases and review of the literature. Possible existence of a new clinical entity originating from the third lineage of lymphoid cells. British journal of haematology. 1990;75(1):49-59.doi:10.1111/j.1365-2141.1990.tb02615.x

2. Song SY, Kim WS, Ko YH, Kim K, Lee MH, Park K. Aggressive natural killer cell leukemia: clinical features and treatment outcome. Haematologica. 2002;87(12):1343-5

3. Ruskova A, Thula R, Chan G. Aggressive Natural Killer-Cell Leukemia: report of five cases and review of the literature. Leuk Lymphoma. 2004;45(12):2427-38.doi:10.1080/10428190400004513

4. Suzuki R, Suzumiya J, Nakamura S, Aoki S, Notoya A, Ozaki S, et al. Aggressive natural killer-cell leukemia revisited: large granular lymphocyte leukemia of cytotoxic NK cells. Leukemia. 2004;18(4):763-70.doi:10.1038/sj.leu.2403262

5. Ryder J, Wang X, Bao L, Gross SA, Hua F, Irons RD. Aggressive natural killer cell leukemia: report of a Chinese series and review of the literature. Int J Hematol. 2007;85(1):18-25.doi:10.1532/ijh97.A10612

6. Ishida F, Ko YH, Kim WS, Suzumiya J, Isobe Y, Oshimi K, et al. Aggressive natural killer cell leukemia: therapeutic potential of L-asparaginase and allogeneic hematopoietic stem cell transplantation. Cancer Sci. 2012;103(6):1079-83.doi:10.1111/j.1349-7006.2012.02251.x

7. Zhang H, Meng Q, Yin W, Xu L, Lie L. Adult aggressive natural killer cell leukemia. Am J Med Sci. 2013;346(1):56-63.doi:10.1097/MAJ.0b013e3182764b59

8. Li Y, Wei J, Mao X, Gao Q, Liu L, Cheng P, et al. Flow Cytometric Immunophenotyping Is Sensitive for the Early Diagnosis of De Novo Aggressive Natural Killer Cell Leukemia (ANKL): A Multicenter Retrospective Analysis. PLoS One. 2016;11(8):e0158827.doi:10.1371/journal.pone.0158827

9. Jung KS, Cho SH, Kim SJ, Ko YH, Kang ES, Kim WS. L-asparaginase-based regimens followed by allogeneic hematopoietic stem cell transplantation improve outcomes in aggressive natural killer cell leukemia. J Hematol Oncol. 2016;9:41.doi:10.1186/s13045-016-0271-4

10. Nicolae A, Ganapathi KA, Pham TH, Xi L, Torres-Cabala CA, Nanaji NM, et al. EBV-negative Aggressive NK-cell Leukemia/Lymphoma: Clinical, Pathologic, and Genetic Features. The American journal of surgical pathology. 2017;41(1):67-74.doi:10.1097/pas.0000000000000735

11. Tang YT, Wang D, Luo H, Xiao M, Zhou HS, Liu D, et al. Aggressive NK-cell leukemia: clinical subtypes, molecular features, and treatment outcomes. Blood Cancer J. 2017;7(12):660.doi:10.1038/s41408-017-0021-z
