# Supplementary material for: Aggressive Natural Killer Cell Leukemia in an Adolescent Patient: A Case Report and Literature Review
Source: Front Pediatr. 2022 May 23;10:829927. doi: 10.3389/fped.2022.829927 (PMC9168658; doi:10.3389/fped.2022.829927)
Supplement: Supplementary file 2 [file Table_2.docx]

**Appendix**

**Table 2 CARE Guidelines for Case Reports**

| **Topic** | **Checklist item description** | **Reported on Line** |
| --- | --- | --- |
| **Title** | The diagnosis or intervention of primary focus followed by the words “case report” | 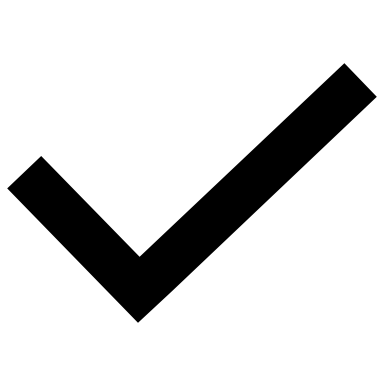 |
| **Key Words** | 2 to 5 key words that identify diagnoses or interventions in this case report, including "case report" | 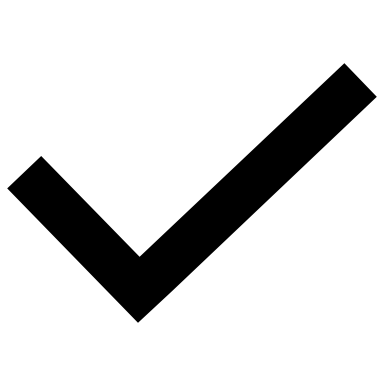 |
| **Abstract (no references)** | Introduction: What is unique about this case and what does it add to the scientific literature? | 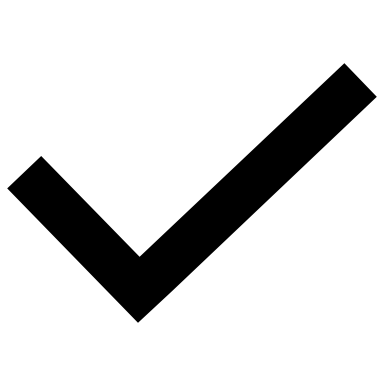 |
|  | Main symptoms and/or important clinical findings | 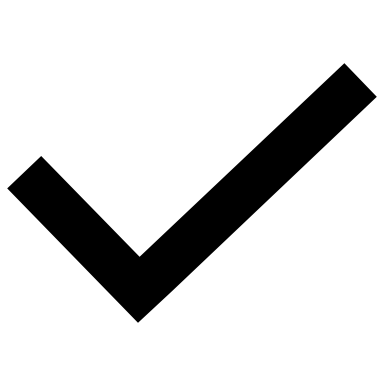 |
|  | The main diagnoses, therapeutic interventions, and outcomes | 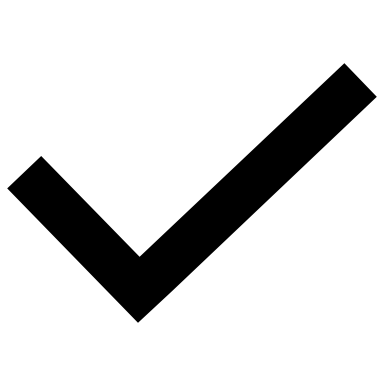 |
|  | Conclusion—What is the main “take-away” lesson(s) from this case? | 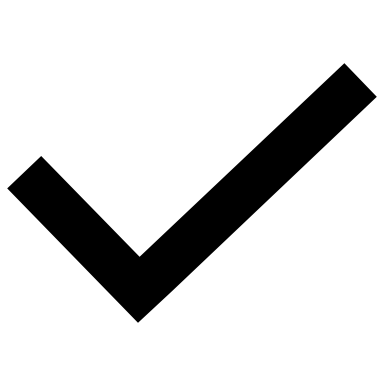 |
| **Introduction** | One or two paragraphs summarizing why this case is unique (**may include** reference**s**) | 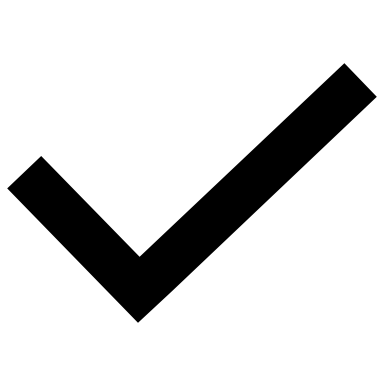 |
| **Patient Information** | De-identified patient specific information | 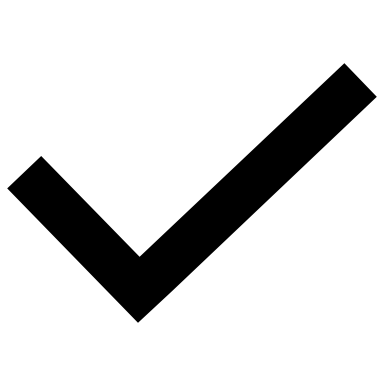 |
|  | Primary concerns and symptoms of the patient | 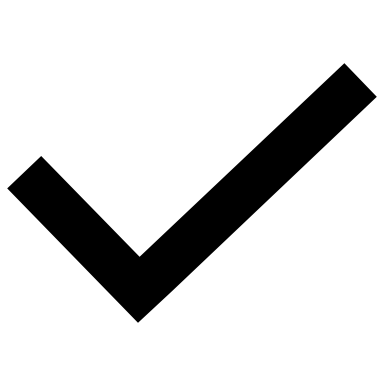 |
|  | Medical, family, and psycho-social history including relevant genetic information | 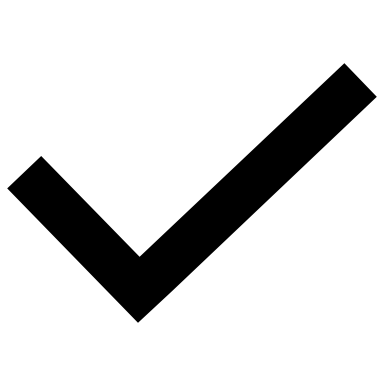 |
|  | Relevant past interventions with outcomes | Not applicable as our patient had no prior medical history and this was the first-time admitting |
| **Clinical Findings** | Describe significant physical examination (PE) and important clinical findings | 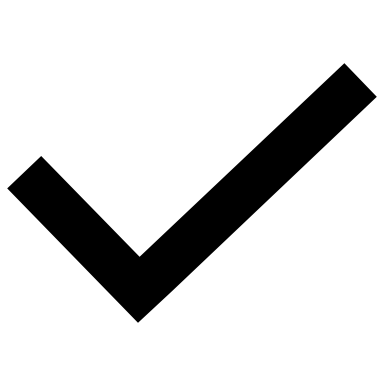 |
| **Timeline** | Historical and current information from this episode of care organized as a timeline (figure or table) | 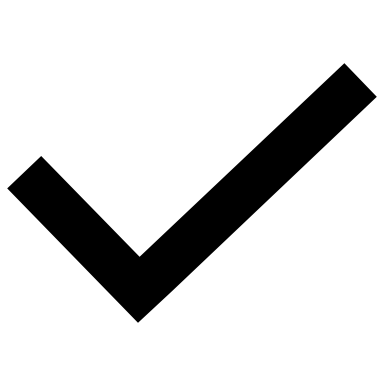 |
| **Diagnostic Assessment** | Diagnostic testing (such as PE, laboratory testing, imaging, surveys) | 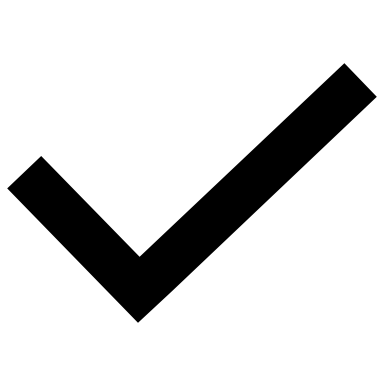 |
|  | Diagnostic challenges (such as access to testing, financial, or cultural) | 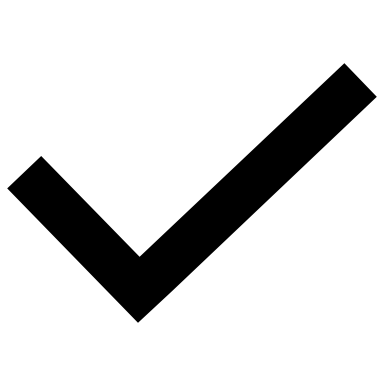 |
|  | Diagnosis (including other diagnoses considered) | 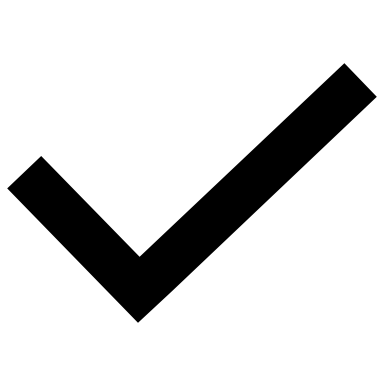 |
|  | Prognosis (such as staging in oncology) where applicable | 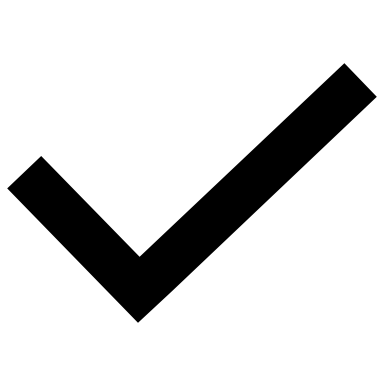 |
| **Therapeutic Intervention** | Types of therapeutic intervention (such as pharmacologic, surgical, preventive, self-care) | 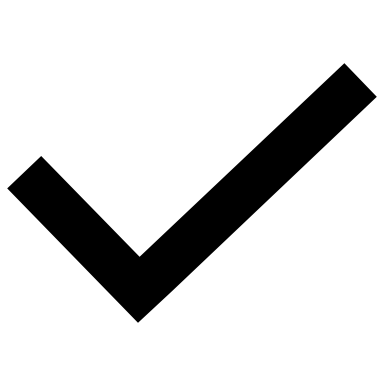 |
|  | Administration of therapeutic intervention (such as dosage, strength, duration) | 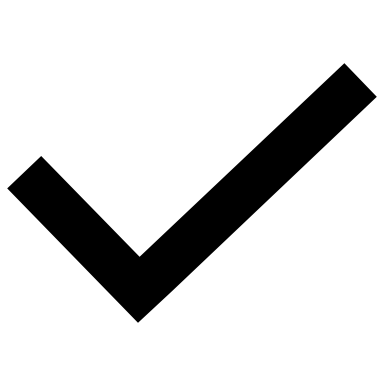 |
|  | Changes in therapeutic intervention (with rationale) | 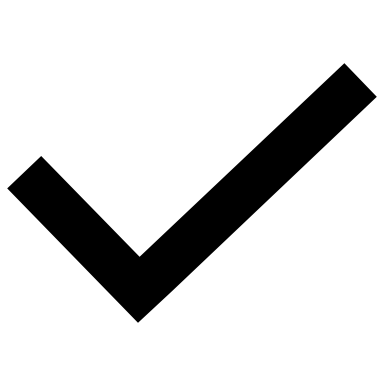 |
| **Follow-up and Outcomes** | Clinician and patient-assessed outcomes (if available) | 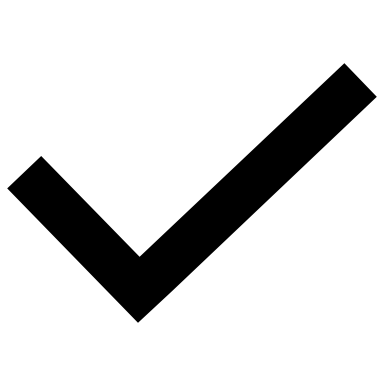 |
|  | Important follow-up diagnostic and other test results | 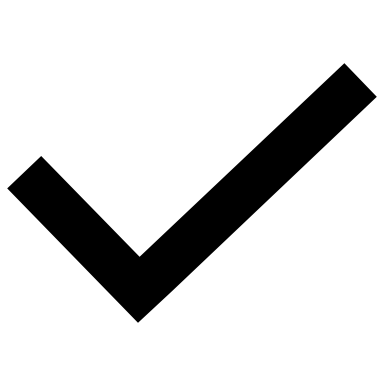 |
|  | Intervention adherence and tolerability (How was this assessed?) | 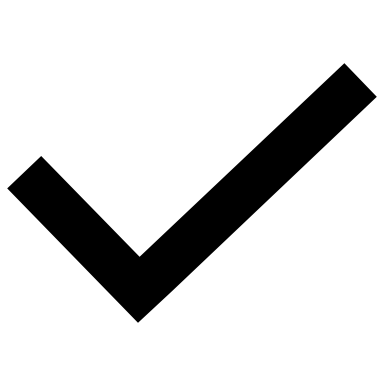 |
|  | Adverse and unanticipated events | 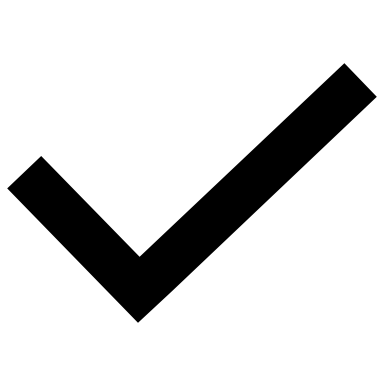 |
| **Discussion** | A scientific discussion of the strengths AND limitations associated with this case report | 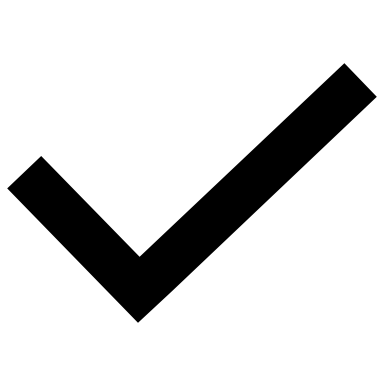 |
|  | Discussion of the relevant medical literature **with references** | 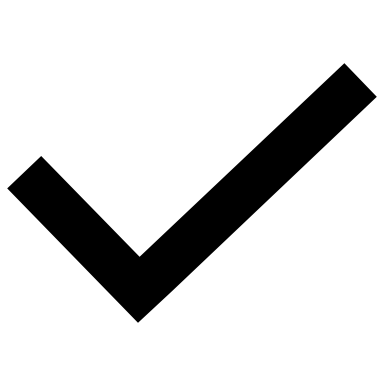 |
|  | The scientific rationale for any conclusions (including assessment of possible causes) | 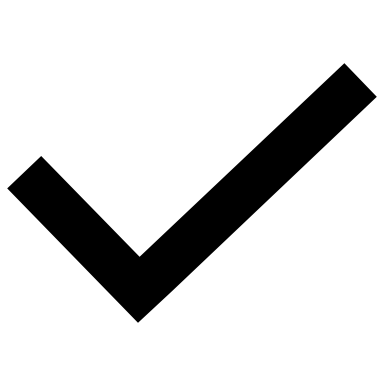 |
|  | The primary “take-away” lessons of this case report (without references) in a one paragraph conclusion | 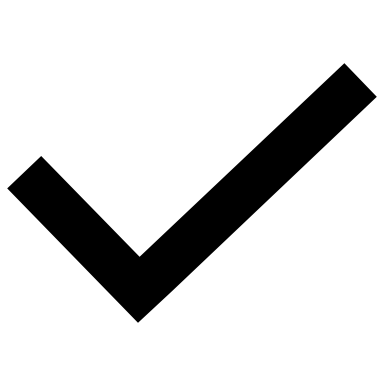 |
| **Patient Perspective** | The patient should share their perspective in one to two paragraphs on the treatment(s) they received | Not applicable for our patient as she is a pediatric patient |
| **Informed Consent** | Did the patient give informed consent? Please provide if requested | 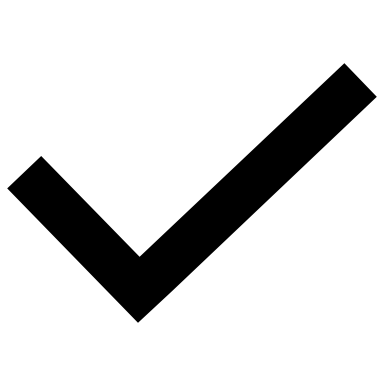 |
